# Intake, digestibility and nitrogen utilization in cattle fed tropical forage and supplemented with protein in the rumen, abomasum, or both

**DOI:** 10.1186/s40104-016-0069-9

**Published:** 2016-02-19

**Authors:** Luana Marta de Almeida Rufino, Edenio Detmann, Daiany Íris Gomes, William Lima Santiago dos Reis, Erick Darlisson Batista, Sebastião de Campos Valadares Filho, Mário Fonseca Paulino

**Affiliations:** Department of Animal Science, Universidade Federal de Viçosa, Av. P.H. Rolfs, s/n°, Viçosa, Minas Gerais 36570-900 Brazil; Department of Animal Science, Universidade Federal Rural da Amazônia, Campus de Parauapebas, C.P. 3017, Bairro Cidade Nova, Parauapebas, Pará 68515-970 Brazil

**Keywords:** Casein, Nitrogen balance, Rumen ammonia nitrogen, Supplementation, 3-methylhistidine

## Abstract

**Background:**

There is little information in the tropics with regard the comparative understanding of how an increased nitrogen supply in the rumen or in the intestines affects efficiency of nitrogen utilization in cattle. This study evaluated the effects of supplementation with nitrogenous compounds in the rumen, abomasum, or both on intake, digestibility and the characteristics of nitrogen utilization in cattle fed tropical forage. Four rumen- and abomasum-fistulated Nellore bulls (227 ± 11 kg) were used. Four treatments were evaluated: control, ruminal supplementation (230 g/d of supplemental protein in the rumen), abomasal supplementation (230 g/d of supplemental protein in the abomasum), and ruminal and abomasal supplementation (115 g/d protein in both the rumen and the abomasum). The basal forage diet consisted of Tifton 85 hay with a crude protein (CP) level of 78.4 g/kg dry matter. Casein was used as a supplement. The experiment was conducted using a 4 × 4 Latin square.

**Results:**

There were no differences between the treatments (*P* > 0.10) with regard to forage intake. The intake and total digestibility of CP increased (*P* < 0.01) with supplementation. The nitrogen balance in the body increased (*P* < 0.01) and muscle protein mobilization decreased (*P* < 0.01) with supplementation, regardless of the supplementation site. The efficiency of nitrogen utilization did not differ among the treatments (*P* > 0.10).

**Conclusions:**

The supplementation of cattle fed tropical forage with protein in the rumen, abomasum, or both similarly increased the nitrogen accretion in animal, which reflects improvements on nitrogen status in animal body.

## Background

It has been established that rumen degradable protein (RDP) constitutes the most important supplement for cattle fed low-quality forages. In studies conducted under tropical conditions, low concentrations of rumen ammonia nitrogen (RAN) have been associated with negative estimates of nitrogen balance in the rumen (NBR), which might increase the mobilization of body proteins to sustain rumen microbial growth [1–4]. Particularly for tropical grasses, a great portion of total nitrogen can be found associated to cell wall, which decreases the degradability of forage crude protein and contributes for the low RAN concentrations [5]. Negative NBR emphasize the importance of available nitrogen in the rumen through urea recycling, which is a byproduct of the nitrogen that is absorbed or mobilized from endogenous sources. Recycled nitrogen may contribute significantly to the supply of nitrogen in the rumen [4, 6].

Once the requirements of the first limiting factor (RDP) have been met, supplying rumen undegradable protein (RUP) could improve the supply of metabolizable protein and decrease the proportion of nitrogen compounds that is recycled to the rumen, thereby increasing the availability of nitrogen for anabolic purposes [7] and reducing the mobilization of endogenous protein [2, 3]. However, RUP supplementation would be a less efficient means to maintain the level of RAN compared with a direct supply of RDP [8]. Recent studies conducted under tropical conditions have shown that protein supplements for ruminants may directly affect the efficiency of conversion of metabolizable protein into net protein [2, 3]. However, there is little information in the tropics with regard the comparative understanding of how an increased nitrogen supply in the rumen or in the intestines affects efficiency of nitrogen utilization in cattle. In this sense, basic research using pure protein sources, such as casein, could be helpful to understand the true effects of protein supplementation on animal metabolism and the utilization efficiency of nitrogen from RDP or RUP in cattle fed tropical forages.

The objective of the current study was to evaluate the effects of supplementation with protein in the rumen, abomasum, or both on intake, digestibility, the rumen dynamics of fibrous compounds, and the efficiency of nitrogen utilization in cattle fed tropical forage.

## Methods

This experiment was carried out at the Department of Animal Science of the Universidade Federal de Viçosa, Viçosa, Brazil. All surgical and animal care procedures were conducted according to the regulations of the Brazilian National Council on the Control of Animal Experimentation (CONCEA).

Four rumen- and abomasum-fistulated Nellore bulls with an initial average body weight (BW) of 227 ± 11 kg were used in this experiment. The animals were kept in individual stalls (2 by 5 m) with concrete floors covered with a rubber layer and equipped with individual feeders and water dispensers. The animals had unrestricted access to mineral mix.

The basal forage consisted of Tifton 85 (*Cynodon* sp.) hay, which had an average crude protein (CP) content of 78.4 g/kg dry matter (DM). The forage was provided *ad libitum* daily at 0600h and 1800h, allowing approximately 100 g/kg in orts.

This study evaluated the following treatments: control (no supplementation); ruminal supplementation, with a daily supply of 230 g of supplemental CP in the rumen; abomasal supplementation, with a daily supply of 230 g of supplemental CP in the abomasum; and ruminal and abomasal supplementation, with a total daily supply of 230 g of supplemental CP (115 g of CP in both the abomasum and the rumen).

The amount of supplement (230 g of CP/d) accounted for approximately 35 % of the daily CP requirements, 55 % of the RDP daily requirements, or 100 % of the RUP daily requirements of a 250-kg Zebu bull with a weight gain of 0.5 kg/d [9]. Casein was used as the source of supplemental protein (pure casein; Labsynth, Diadema, SP, Brazil). This protein source was used as the RDP and RUP supplement because of its high-protein content, it is readily degraded in the rumen and/or digested in the small intestine, and to avoid confounding effects by using different protein sources in the rumen and abomasum.

The experiment consisted of four 24-day experimental periods. An 8-day interval was applied between experimental periods to reduce the residual effects of the treatments. The first 14 d of each period were used for treatment adaptation. Prior to the experiment, the animals were adapted to the experimental conditions and basal forage for 14 d.

The total supplement was separated into two portions of equal weight and supplied to the animals when the forage was offered (0600h and 1800h). The ruminal supplement was packaged in paper bags and placed directly into the rumen of the animals. The casein for the abomasal supplementation was diluted in saline solution (NaCl, 9 g/L). The lids of the abomasal cannulas were fitted with approximately 15 cm of polyethylene tubing to form external valves. The supplement was infused into the abomasum through these valves.

The samples were collected between the 15^th^ and 24^th^ d of each experimental period. The forage supplied from the 15^th^ to 18^th^ d and the orts obtained from the 16^th^ to 19^th^ d were used to measure the voluntary intake.

Fecal grab samples were taken from the rectum of the animals between the 16^th^ and 19^th^ d of each experimental period according to the following schedule: 16^th^ day – 0600h and 1400h, 17^th^ day – 0800h and 1600h, 18^th^ day – 1000h to 1800h, 19^th^ day – 1200h and 2000h. Samples of abomasal digesta were simultaneously collected with the fecal samples. These samples were oven-dried (60 °C) and processed in a knife mill (1- and 2-mm; Model 4, Thomas Wiley Co., Swedesboro, NJ).

Total urine collection was performed on the 20th day of each experimental period. Collecting funnels were attached to the animals to direct the urine into polyethylene flasks that were kept cool in a polystyrene cooler with ice. The collections began at 0600h and lasted 24 h. At the end of the collection period, the urine was measured, and two 50-mL aliquots were collected for analyses. The first aliquot was used to assess nitrogen, urea, and creatinine contents. The second aliquot was used to quantify the content of 3-methyl histidine (3-MH).

On the 21st day, blood samples were taken from the animals at 0600h, 1200h, 1800h and 2400h. directly from the jugular vein using vacuum tubes with either coagulation accelerator gel (BD Vacutainer®, SST II Advance, Franklin Lakes, NJ) or coagulation inhibitor gel (BD Vacutainer® K2, Franklin Lakes, NJ). The samples collected with coagulation accelerator were centrifuged (2,700 × g for 20 min) to separate the serum, and their urea and creatinine levels were evaluated. The samples obtained with coagulation inhibitor were refrigerated (4 °C). At the end of the collection period, the samples were combined for each animal to assess the concentration of free amino acids (AA).

Rumen content samples were also collected at 0600h, 1200h, 1800h and 2400h during the 21^st^ d of each experimental period to isolate microorganisms using the technique described by Cecava et al. [10]. In addition, ruminal aliquots were taken to evaluate pH, and concentrations of RAN and volatile fatty acids (VFA; acetate, propionate, and butyrate). These samples were manually collected at the liquid–solid interface of the rumen mat, filtered through a triple cheesecloth layer, and subjected to pH evaluation (potentiometer TEC-3P-MP, Tecnal, Piracicaba, SP, Brazil). Next, a 40-mL aliquot was separated, fixed with 1 mL of H_2_SO_4_ (1:1), and frozen (−20 °C) for RAN concentration analysis. A second 20-mL aliquot was fixed with 5 mL of a meta-phosphoric acid solution (250 g/L) and kept at −20 °C for subsequent assessment of the VFA concentration.

The rumen evacuation procedure was performed on the 22^nd^ and 24^th^ d to quantify the resident mass and the rates of passage and degradation of the fibrous material. The rumen content was removed at 1000h (4 h after the morning feeding) and 0600 (before the morning feeding) on the aforementioned days, respectively. The collected material was packed in a polyethylene container and weighed. The material was stirred by hand and an aliquot of approximately 50 g/kg was removed. The remaining material was returned to the rumen of the animals. The samples were oven-dried (60 °C) and processed in a knife mill (1- and 2-mm).

Subsequently, the samples of hay, orts, feces, abomasal digesta, and ruminal contents (the samples collected from ruminal evacuation) were pooled per animal and experimental period.

Chemical analyses were performed on the samples that were processed to pass through a 1-mm sieve. The contents of DM (method INCT-CA no. G-003/1), organic matter (OM; method INCT-CA no. M-001/1), CP (Kjeldahl procedure; method INCT-CA no. N-001/1), neutral detergent fiber corrected for ash and protein (NDFap; using a heat-stable α-amylase, omitting sodium sulfite and correcting for residual ash and protein; method INCT-CA no. F-002/1), neutral detergent insoluble protein (method INCT-CA no. N-002/1), and acid detergent lignin (method INCT-CA no. F-005/1) were quantified according to the standard analytical procedures of the Brazilian National Institute of Science and Technology in Animal Science (INCT-CA; Table 1) [11]. The casein samples were evaluated for DM, OM, and CP contents according the methods described above (Table 1).

**Table 1:** Chemical composition of hay and casein

Item	Hay^b^	Casein
Dry matter (DM), g/kg as fed	907 ± 8.2	892
Organic matter, g/kg DM	933 ± 0.6	973
Crude protein, g/kg DM	78 ± 0.3	899
NDFap^a^, g/kg DM	730 ± 7.8	-
NDIP^a^, g/kg crude protein	360 ± 78	-
Lignin, g/kg DM	58 ± 0.7	-
iNDF^a^, g/kg DM	391 ± 5.2	-

^a^ *NDFap* NDF assayed with a heat-stable alpha-amylase and corrected for contaminant ash and protein; *NDIP* neutral detergent insoluble protein; *iNDF* indigestible neutral detergent fiber

^b^Mean ± standard error

Fecal excretion and abomasal flow were estimated by using the indigestible NDF (iNDF) as internal marker. The samples of hay, orts, feces, abomasal digesta, and ruminal contents, processed by passing through a 2-mm screen sieve, were evaluated with regard to iNDF content using F57 bags (Ankom Technology Corp., Macedon, NY) and an *in situ* incubation procedure for 288 h (method INCT-CA no. F-008/1) [11]. Importantly, the supplement infused into the abomasum was not considered in the ruminal digestibility and outflow calculation; rather, it was only used to calculate the intestinal digestibility coefficients.

The rates of intake and ruminal passage of NDF were estimated by the ratio of NDF intake and abomasum flow on the rumen mass of NDF, respectively. The degradation rate of NDF was obtained as the difference between the rates of intake and passage [12].

The RAN concentration was quantified using the colorimetric technique described by Detmann et al. [11] (method INCT-CA no. N-006/1). The concentrations obtained at different sampling times were combined for each animal and period in order to obtain a single value that represented the average daily RAN concentration. The rumen pH values were combined in a similar way.

The VFA concentration was evaluated on pooled rumen fluid samples composed of proportional sample volumes for each collection (per animal and period) and evaluated using HPLC (Shimadzu chromatograph, Model SPD-10A VP) with a reverse phase column (using a mobile phase of orthophosphoric acid in water, 10 mL/L) and a UV detector at a wavelength of 210 nm.

The samples of ruminal microorganisms were analyzed for CP, as described for feed samples, and for purine bases [13]. The purine bases were used to assess the microbial concentrations in the abomasal digesta based on the N_RNA_:N_total_ ratio in rumen microorganisms.

The urine samples were analyzed for nitrogen content as described for the CP analysis of the feed samples. The urea and creatinine concentrations in the urine and blood serum were evaluated using the enzymatic-colorimetric (K047, Bioclin Co., Belo Horizonte, MG, Brazil) and alkaline picrate (K016, Bioclin Co., Belo Horizonte, MG, Brazil) methods, respectively. The 3-MH in urine and the total free AA in blood were obtained using the HPLC techniques described by Jones et al. [14] and Pitta et al. [15], respectively.

The urea nitrogen filtered in the kidneys and the fractional excretion of urea nitrogen were calculated from the following equations:

1 $$ UNFK=\frac{UEC}{SC}\times SUN $$

2 $$ FEUN=\frac{EUN}{UNFK} $$

where UNFK is the urea nitrogen filtered in the kidneys (g/d), UEC is the urinary excretion of creatinine (g/d), SC is the average concentration of serum creatinine (mg/dL), SUN is the average concentration of serum urea nitrogen (mg/dL), FEUN is the fractional excretion of urea nitrogen (g/g), and EUN is the urinary excretion of urea nitrogen (g/d).

The experiment was carried out and analyzed according to a 4 × 4 Latin square design balanced for residual effects with four treatments (fixed effect), four animals (random effect), and four experimental periods (random effect).

All of the statistical procedures were carried out using the MIXED procedure of SAS 9.3. Due to the high probability of type II error, we adopted α = 0.10. When necessary, the treatment means were compared using protected Fisher’s least significant difference. The data from one purine base concentration in a microbial sample and one creatinine concentration in a blood sample were lost during the analysis.

## Results

There were no differences among treatments with regard to the intake of DM (*P* > 0.22), forage (*P* > 0.30), OM (*P* > 0.22), neutral detergent fiber (NDF; *P* > 0.34), iNDF (*P* > 0.22), and digested NDF (*P* > 0.36) (Table 2).

**Table 2:** Effects of supplementation with protein in different sites on voluntary intake in cattle fed tropical forage

	Supplementation site^d^					
Item^c^	Control	R	A	R + A	SEM	*P*-value
	kg/d					
DM	5.05	5.60	5.13	5.12	0.48	0.229
DMF	5.05	5.35	4.88	4.87	0.48	0.307
OM	4.72	5.25	4.81	4.80	0.45	0.226
CP	0.403^b^	0.648^a^	0.611^a^	0.613^a^	0.040	<0.001
NDFap	3.65	3.87	3.54	3.54	0.33	0.348
iNDF	1.93	2.05	1.87	1.84	0.19	0.222
DOM	2.22^b^	2.60^a^	2.31^b^	2.40^ab^	0.22	0.060
DNDF^)^	1.94	2.01	1.84	1.87	0.17	0.362
	g/kg body weight					
DM	20.4	22.4	20.8	20.7	1.2	0.263
OM	19.1	21.0	19.5	19.4	1.1	0.260
NDFap	14.8	15.5	14.3	14.3	0.8	0.337
iNDF	7.8	8.2	7.6	7.4	0.5	0.273

^a, b^within a row, means without a common superscript differ (*P* < 0.10)

^c^ *DM* dry matter; *DMF* dry matter from forage; *OM* organic matter; *CP* crude protein; *NDFap* neutral detergent fiber assayed with a heat-stable alpha-amylase and corrected for contaminant ash and protein; *iNDF* indigestible neutral detergent fiber; *DOM* digested organic matter; *DNDF* digested neutral detergent fiber

^d^Control = without supplementation; R = ruminal supplementation; A = abomasal supplementation

The CP intake increased with supplementation (*P* < 0.01), but no differences were found between the supplementation sites (*P* > 0.10). The mean CP intakes were 0.403 kg/d and 0.624 kg/d for the control and supplementation treatments, respectively (Table 2). Rumen supplementation increased the intake of digested OM (DOM) compared with that of the control and with that of the abomasal supplementation treatments (*P* < 0.10). An intermediate value of DOM intake was observed for the rumen/abomasal supplementation (Table 2).

The results of the total digestibility coefficient of CP were similar to those observed for the CP intake (Table 3). Supplementation increased total digestibility of CP from 0.475 g/g to 0.653 g/g on average. No differences were found among treatments for the total digestibility of OM (*P* > 0.14) and NDF (*P* > 0.89) (Table 2).

**Table 3:** Effects of supplementation with protein in different sites on total, ruminal, and intestinal digestibilities (g/g) in cattle fed tropical forage

	Supplementation site ^e^					
Item^f^	Control	R	A	R + A	SEM	*P*-value
	Total					
OM	0.468	0.495	0.485	0.502	0.017	0.142
CP	0.475^b^	0.656^a^	0.639^a^	0.664ª	0.023	<0.001
NDFap	0.531	0.519	0.523	0.532	0.019	0.898
	Ruminal^g^					
OM	0.300	0.324	0.277	0.284	0.031	0.265
CP	−0.221^b^	0.123^a^	−0.987^c^	−0.284^b^	0.139	<0.001
NDFap	0.502^c^	0.512^bc^	0.549^a^	0.528^ab^	0.024	0.041
	Intestinal^g^					
CP	0.561^d^	0.607^c^	0.772^a^	0.718^b^	0.026	<0.001

^a, b, c, d^within a row, means without a common superscript differ (*P* < 0.10)

^e^Control = without supplementation; R = ruminal supplementation; A = abomasal supplementation

^f^OM, organic matter; CP, crude protein; NDFap, neutral detergent fiber assayed with a heat-stable alpha-amylase and corrected for contaminant ash and protein

^g^Ruminal and intestinal digestibilities were calculated as the fraction of the mass that entered the digestion site

No differences were observed among treatments with regard to the ruminal digestibility of OM (*P* > 0.26; Table 3). The rumen digestibility of CP was higher (*P* < 0.10) with ruminal supplementation than for the other treatments and was lowest for the abomasal supplementation. Ruminal CP digestibility was intermediate from the control and supplementation in the rumen and abomasum. Importantly, the CP rumen digestibility was positive in the ruminal supplementation group and negative for the other treatments (Table 3).

Abomasal and ruminal/abomasal supplementation resulted in higher NDF rumen digestibility compared with the control (*P* < 0.10), with an intermediate value observed for ruminal supplementation (Table 3).

The CP intestinal digestibility differed across all of the treatments (*P* < 0.10). The following treatments are presented in descending order: abomasal supplementation, ruminal/abomasum supplementation, rumen supplementation, and control (Table 3).

Treatments did not affect ruminal NDF (*P* > 0.33) and iNDF (*P* > 0.82) mass; their mean values were 10.5 and 8.5 g/kg BW, respectively. In addition, no differences were observed among treatments with regard to the rates of intake (*P* > 0.23), degradation (*P* > 0.33), and passage of NDF (*P* > 0.14) (Table 4).

**Table 4:** Effects of supplementation with protein in different sites on the resident mass of fiber in the rumen, the fractional rates of NDF rumen dynamics, and on the characteristics of ruminal fermentation in cattle fed tropical forage

	Treatments^c^					
Item^d^	C	R	A	R + A	SEM	*P*-value
Resident mass in the rumen						
NDF, g/kg BW	10.7	10.0	11.8	9.6	1.3	0.337
iNDF, g/kg BW	8.4	8.0	9.2	8.3	1.0	0.821
NDF rumen dynamics						
ki, /h	0.060	0.071	0.052	0.063	0.008	0.238
kp, /h	0.029	0.034	0.023	0.030	0.003	0.149
kd, /h	0.030	0.037	0.029	0.034	0.005	0.338
Ruminal characteristics						
RAN, mg/dL	4.15^b^	13.66^a^	5.56^b^	8.22^ab^	1.91	0.078
pH	6.83	6.75	6.79	6.72	0.12	0.914
VFA, mmol/dL	5.321	5.396	5.731	5.940	0.279	0.256
Acetate, mol/mol	73.71	73.40	75.25	74.06	0.87	0.489
Propionate, mol/mol	18.76	19.08	17.74	19.32	0.79	0.559
Butyrate, mol/mol	7.53	7.53	7.01	6.69	0.32	0.230
A:P	3.96	3.86	4.29	3.84	0.22	0.501

^a, b^within a row, means without a common superscript differ (*P* < 0.10)

^c^Control = without supplementation; R = ruminal supplementation; A = abomasal supplementation

^d^ *NDF* neutral detergent fiber; *iNDF* indigestible neutral detergent fiber; *ki, kp and kd* rates of intake, passage and degradation; *RAN* rumen ammonia nitrogen; *VFA* volatile fatty acids; *A:P* acetate to propionate ratio

The ruminal pH did not vary across treatments (*P* > 0.91), and an average value of 6.78 was observed. However, higher RAN concentrations were found for the rumen supplementation group compared with the control and the abomasum supplementation groups (*P* < 0.10). These latter groups did not differ from each other (*P* > 0.10). Rumen/abomasal supplementation resulted in an intermediate RAN concentration (Table 4).

There were no differences among the treatments regard to the VFA concentration (*P* > 0.25), the average value of which was 5.60 mmol/dL. In addition, the acetate (*P* > 0.48), propionate (*P* > 0.55), and butyrate (*P* > 0.23) molar ratios, as well as the acetate:propionate ratio (*P* > 0.50), did not differ across treatments (Table 4).

The SUN concentration (Table 5) was higher with ruminal supplementation compared with ruminal/abomasum supplementation and control (*P* < 0.10). The average SUN concentration obtained with abomasal supplementation occupied an intermediate position compared with the other supplementation types. No differences were found across the treatments with regard to the concentration of AA in the blood (*P* > 0.48; Table 5).

**Table 5:** Effects of supplementation with protein in different sites on the characteristics of nitrogen utilization in the animals in cattle fed tropical forage

	Supplementation site^e^					
Item^d^	Control	R	A	R + A	SEM	*P*-value
SUN, mg/dL	7.29^c^	17.16^a^	13.46^ab^	13.39^b^	1.30	0.029
BAA, μmol/mg creatinine	113	123	129	119	12	0.485
Nitrogen intake, g/d	64.5^b^	103.8^a^	97.8^a^	98.0^a^	6.4	<0.001
Fecal nitrogen, g/d	34.0	36.0	35.5	33.4	4.0	0.611
Urinary nitrogen, g/d	26.3^b^	48.5^a^	40.4^a^	46.3^a^	4.2	0.010
Nitrogen balance, g/d	4.1^b^	19.3^a^	22.0^a^	18.3^a^	6.6	0.062
NBR, g/d	−14.4^c^	12.2^a^	−23.2^c^	−2.0^b^	7.5	0.005
ENU						
g retained nitrogen/g of ingested nitrogen	0.04	0.18	0.22	0.18	0.05	0.165
g retained nitrogen /g of nitrogen absorbed in the intestines	0.07	0.27	0.35	0.27	0.04	0.402
PDI, g/d	280^c^	347^bc^	535^a^	416^b^	45	0.003
3-MH, mg/g of creatinine	50.8^a^	20.6^b^	29.6^b^	31.0^b^	13.1	0.077
UEUN, g/d	13.4^b^	28.9^a^	25.9ª	30.1^a^	2.9	0.002
UFK, g/d	42.9 ± 6.5^b^	60.9 ± 5.6^a^	43.9 ± 5.6^b^	55.0 ± 5.6^a^	---	0.062
FUEUN, g/g	0.35 ± 0.09	0.48 ± 0.08	0.63 ± 0.08	0.55 ± 0.08	---	0.121
NMIC, g/d	53.1 ± 13.0	61.2 ± 11.1	61.9 ± 13.0	58.8 ± 11.1	---	0.932

^a, b, c^within a row, means without a commom superscript differ (*P* < 0.10)

^d^ *SUN* serum urea nitrogen; *BAA* amino acids in blood; *NBR* nitrogen balance in the rumen; *ENU* efficiency of nitrogen utilization; *PDI* protein digested in the intestine; *3-MH* urinary excretion of 3-methyl histidine; *UEUN* urinary excretion of urea nitrogen; *UFK* urea nitrogen filtered in the kidneys; *FUEUN* fractional excretion of urea nitrogen; *NMIC* ruminal production of microbial nitrogen compounds

^e^Control = without supplementation; R = ruminal supplementation; A = abomasal supplementation

Nitrogen intake and urinary excretion increased with protein supplementation, regardless of the supplementation site (*P* < 0.10). However, the nitrogen fecal excretion did not vary among the treatments (*P* > 0.61). Similarly, the apparent nitrogen balance (NB) increased with nitrogen supplementation compared with the control treatment, regardless of the supplementation site (*P* < 0.10; Table 5). However, in spite of the great numerical differences between control and supplemented treatments, no differences in the efficiency of nitrogen utilization were observed among the treatments (*P* > 0.16).

The highest estimate of NBR was observed with rumen supplementation (*P* < 0.10), followed by ruminal/abomasal supplementation, abomasum supplementation, and the control. No differences were observed between the latter two treatments (*P* > 0.10). Importantly, only ruminal supplementation resulted in positive NBR (Table 5).

The treatments affected the amount of CP digested in the intestines (PDI; *P* < 0.01). Larger amounts of PDI were observed in the abomasal supplementation group compared with the ruminal/abomasal supplementation and the control treatments. The ruminal supplementation group held an intermediate position between these groups (Table 5).

The control treatment exhibited greater urinary excretion of 3-MH compared with the supplement treatments (*P* < *0*.10), which did not differ from each other (*P* > 0.10). The urinary excretion of urea nitrogen was higher for the supplementation groups than for the control group (*P* < 0.10). In addition, ruminal and ruminal/abomasal supplementation increased the urea filtered in the kidneys (*P* < 0.10) compared with the other treatments. Supplementation did not alter the fractional excretion of urea nitrogen (*P* > 0.12). However, supplementation resulted in a 60 % higher fractional excretion of urea nitrogen compared with the control (Table 5). The ruminal synthesis of microbial nitrogenous compounds did not differ among the treatments (*P* > 0.93), with an average value of 58.7 g/day (Table 5).

## Discussion

Nitrogen supplementation for animals fed low-quality forage favors the growth of fibrolytic bacteria and increases the ruminal degradation and voluntary intake of fiber, as well as the energy extraction from forage fiber [16]. Specifically, supplementation with nitrogenous compounds in the abomasum of animals fed low-quality forage could stimulate intake via nitrogen recycling, albeit with less intensity compared with rumen supplementation [6, 8]. However, positive effects on the voluntary intake of forage were not observed for any supplementation site in the present study (Table 2).

The results obtained here differ from those reported by several authors who found increases in the voluntary intake of low-quality forage in cattle supplemented with nitrogenous compounds in the rumen [1, 17–19] or in the abomasum [6, 8, 20].

The lack of changes on forage and NDF intake in the response to nitrogen supplementation might be associated with the content of CP in the basal forage (Table 1). The previously cited authors studied forages with CP contents that were typically below 60 g/kg DM. The average dietary CP in the control treatment, calculated as the ratio between CP and DM intake, was 79.8 g/kg. Nitrogen does not positively affect voluntary forage intake when the dietary CP is above 70–80 g/kg DM [3, 21].

The stimulation of low-quality forage intake via nitrogen supplementation is usually associated with a reduction in the physical constraints to intake [16, 22]. In this context, nitrogen supplementation could increase the degradation rate of NDF, which concomitantly increases the passage rate of non-degraded and indigestible fiber from the rumen, increasing forage intake [15].

However, supplementation did not alter the ruminal dynamics of NDF (Table 4). This result might be also due to the CP content of the basal forage, which was above the minimum level required to support fibrolytic activity in the rumen [23]. When the removal of fiber residues from the rumen is limited, animals can increase their rumen volume to accommodate a greater mass of resident fiber [24]. With this adaptation, and keeping the passage and fractional degradation rates constant, the animal is able to increase the amount of degraded fiber. However, no effect was observed with regard to the rumen fiber mass (Table 4). Therefore, considering the results regarding voluntary intake and fiber dynamics, it can be stated that the CP content of the basal forage was adequate to support ruminal function.

Although supplementation increased the NDF digestibility in the rumen, the observed effect was small, which resulted in no differences among treatments with regard NDF intake and total digestibility, and VFA concentration (Tables 2, 3, and 4). The stimulation observed with supplementation most likely resulted from some improvement in the availability of RAN (Table 4). The “protein effect” can explain the low rumen digestibility coefficient with rumen supplementation. This result corresponds to an increase in the competition for essential substrates between fibrolytic and non-fibrolytic species when the supplements contain true protein [25]. This “protein effect” can occur simultaneously with the nitrogen stimulation of fibrolytic activity in low-quality forage [26]. Therefore, the similarity between control and ruminal supplementation treatments regarding the ruminal digestibility of NDF may be a result of a counterbalancing of the nitrogen stimulus and the “protein effect.” This theory also explains the intermediate position achieved by the ruminal/abomasum supplementation group (Table 3).

The only between-treatment difference found with regard to the total digestion was the CP digestibility. Specifically, these values were higher in the supplemented animals compared with the non-supplemented animals (Table 3). This result occurred as a result of the increased CP intake provided by supplementation (Table 2) because, for the dietary non-fibrous components, such as the CP derived from casein, the apparent digestibility coefficient is proportional to the intake [21]. A similar result was observed for the intestinal digestibility of CP, which was especially high when the casein was infused into the abomasum (Table 3). The assessment of the amount of apparently digested CP in the intestines using the Lucas test approach revealed a true intestinal digestibility of CP of 0.931 g/g [CI(β)_0.90_: 0.838 ≤ β ≤ 1.024]. Considering that there was no difference among the treatments with regard to the fecal excretion of nitrogen (Table 5), this estimate suggests that the protein supplement was almost completely digested in the intestines.

The intake of DOM increased with supplementation in the rumen. However, no between-treatment differences were found with regard to the intake of digested NDF (Table 2). The increased intake of DOM is therefore attributed solely to the effects of the non-fibrous portion of the diet. Casein is estimated to have a ruminal digestion greater than 0.80 g/g [27], whereas its intestinal digestibility coefficient was 0.93 g/g. Therefore, casein supplied in the rumen should result in a higher total digestion compared to casein supplied in the abomasum, which would explain the greater intake of digested OM in the animals that received ruminal supplementation (Table 2).

The most prominent effects of supplementation in the present study were observed on the metabolism and the nitrogen accretion in animal body, as other authors have shown in tropical conditions [2–4, 28].

The main effect of supplementation can be associated with an increase in NB (Table 5), which would represent an increase in weight gain. Although similar amounts of protein were supplied to the supplemented animals, the pathways through which these supplements affect protein accretion in the body seem differ because the way in which supplemental nitrogen is utilized could depend on the supplementation site (i.e., the rumen or the intestines).

Due to the high degradability of casein in the rumen, little of the ruminal protein supplement would reach the intestines to be digested. Considering that the ruminal degradation coefficient is 0.80 g/g [27] and the intestinal digestibility coefficient was 0.93 g/g, the additional protein mass (230 g CP/d) should provide only 42.8 g of additional PDI. This result explains the small increase in PDI that was observed with ruminal supplementation (Table 5). Considering the efficiency of utilization of the absorbed nitrogen (0.27 g/g; Table 5), that additional PDI should increase NB by approximately 1.8 g/d, which is equivalent to approximately 11 % of the increase in NB in relation to the control treatment. Therefore, the probable escape of casein to the small intestine cannot explain the effects of ruminal casein supplementation on NB. It should be emphasize that there was no effect of supplementation on ruminal synthesis of microbial nitrogen (Table 5) and an increase in metabolizable protein supply from microbial protein did not occur.

The most prominent effect of the ruminal supplementation was the increase in RAN concentration (Table 4), which means an overall improvement in nitrogen availability in the animal gastrointestinal tract and also for the animal metabolism. The RAN concentration observed in non-supplemented animals (4.15 mg/dL) was below that reported by Detmann et al. [22] for an equilibrium between the inflow and outflow of nitrogen in the rumen of animals fed tropical forage (approximately 8.4 mg of RAN/dL), which resulted in negative NBR values (Table 5).

The overall effects of nitrogen availability on ruminant metabolism have been associated with a better adequate protein or nitrogen status [7, 29]. Theoretically, the nitrogen status defines the availability of different nitrogenous compounds in both quantity and quality for all required physiological functions in animal metabolism, including the functions associated with the metabolism of other compounds such as energy compounds [7].

Taking into account the theoretical concept of nitrogen status, it could be realized that the nitrogenous compounds would be used in different metabolic functions following an order of priority to the animal: survival, maintenance, and production [7]. Thereby, a positive nitrogen accretion in the animal body or products only will take place after supplying the higher priority demands for nitrogenous compounds.

One of the possible high-priority metabolic functions is the recycling of nitrogen to the gastrointestinal tract. Such a statement seems to be plausible because a continuous supplying on nitrogen for microbial growth in the rumen must be seen as a strategy for animal survival [21, 30]. Under a nitrogen deficiency, the animal is able to decrease urinary nitrogen excretion and increase the fraction of dietary nitrogen that is recycled to the rumen [4, 31]. In fact, despite the absence of a significant difference (*P* > 0.12), the fractional excretion of urea nitrogen in the control treatment was approximately 71 % of that observed in the ruminal supplementation treatment (Table 5). This result indicates that a smaller percentage of the circulating urea was eliminated in the urine, and more prominent fraction was directed for reuse (e.g., for recycling) when supplement was not provided.

When nitrogen deficiency becomes more severe, the animal can increase the myofibrillar protein mobilization to sustain the mass of recycling nitrogen [4, 32]. This was observed in this study through urinary excretion of 3-MH, which was decreased when supplement was provided (Table 5).

The 3-MH is an AA formed from the methylation of histidine after its inclusion in the muscle proteins (i.e., actin and myosin). When muscle proteins are degraded, the 3-MH cannot be reused for protein synthesis and is excreted in the urine [33]. Thus, the 3-MH urinary excretion is a marker for the degradation of muscle protein that, in turn, can be used for other physiological or metabolic functions, such as the supply of higher priority nitrogen demands. The control treatment therefore increased the mobilization of muscle protein by approximately 146 % compared with the ruminal supplementation treatment (Table 5). Similarly, Pitta et al. [15] found a reduction in the plasma concentration of 3-MH by providing a protein supplement to sheep fed low-quality forage.

Importantly, 3-MH urinary excretion was strongly and negatively correlated with SUN (r = −0.892; *P* < 0.01) and RAN (r = −0.693; *P* < 0.09) concentrations, which indicates that muscle protein mobilization is negatively associated with nitrogen availability or nitrogen status.

Considering a normal feeding situation, without any prominent dietary nitrogen deficiency, the amount of nitrogen that is recycled through rumen wall seems to be relatively constant [17]. Therefore, there will be lesser nitrogen accretion in the animal body under low nitrogen contents in the diet because a greater percentage of the ingested nitrogen will be directed to recycling and, as a consequence, a lower percentage of nitrogen will be available for production [7].

The pattern here observed for NBR can be used to give some support for the assumptions about metabolic priorities of nitrogen. Several estimates of negative NBR has been obtained in experiments carried out in the tropics, and the main factor to influence that is the nitrogen availability in the diet [7]. This pattern highlights that nitrogen flow to abomasum can be greater than nitrogen intake in several occasions. In these cases, there is a more significant dependency on recycling events to provide adequate nitrogen supplying to the rumen. Then, the animal will decrease the efficiency of utilization of metabolizable protein for gain (decreased anabolism as supported by the lower numerical efficiency of nitrogen utilization, Table 5) and sometimes also increase the breakdown of muscle protein to supply the nitrogen demands of higher priority (increased catabolism as supported by greater 3-MH excretion, Table 5).

The availability of RAN increased with rumen supplementation (Table 4), making the NBR positive, reflecting improvement in nitrogen status in the animal body. In this way, there was an increase in the accretion of absorbed nitrogen (Table 5). Costa et al. [2] observed similar pattern by providing nitrogen compounds that were highly degradable in the rumen to cattle grazing tropical forage (99 g CP/kg DM).

On the other hand, the direct effect of abomasal supplementation was based on the increased availability of AA absorbed in the small intestine, which can be seen by the greater amount of PDI compared with the control (Table 5). This metabolizable protein supply can be directly incorporated into tissue, thereby increasing the NB (Table 5). Importantly, the efficiency of utilization of absorbed nitrogen and NB were similar for ruminal and abomasal supplementation groups (Table 5).

The largest mass of available AA in the small intestine directly increases the nitrogen status in the animal metabolism by supplying the requirements for tissue synthesis and providing nitrogen to the higher demand events without major mobilizations of muscle protein. The reduction of 3-MH excretion observed in the abomasal supplementation group indirectly confirmed this effect (Table 5).

According to Bandyk et al. [8] and Wichersham et al. [6, 20], supplementation with protein sources that are not degradable in the rumen can increase the supply of RAN through nitrogen recycling, although less efficiently than supplementation with RDP sources. The increased SUN concentration, which is mediated by the greater availability of nitrogen in the intestines, favors this increase in recycling, as was observed in the present study (Table 5).

An increased SUN concentration can increase the difference between the concentrations in the blood and rumen and increases urea transfer [20]. This result should increase the pool of RAN [6, 20]. Wickersham et al. [20] found that the proportion of microbial nitrogen produced from recycled nitrogen increased from 0.31 to 0.58 g/g when cattle fed low-quality forage were supplemented with nitrogenous compounds in the small intestine.

The RAN concentration after abomasal supplementation was increased by only 1.41 mg/dL over the control treatment, and this increase was not significant (*P* > 0.10). This result contradicts the studies described above. However, abomasal supplementation decreased the rumen digestibility of CP and, consequently, the NBR (Tables 3 and 5). Considering that the supplemental nitrogen was not used to estimate ruminal digestibility when supplement was supplied in the abomasum and that forage intake was constant among treatments (Table 2), the only likely cause for the decreased NBR under abomasal supplementation is an increase in the amount of nitrogen coming from recycling. It can therefore be inferred that abomasal supplementation increased the pool of available nitrogen in the rumen, although less efficiently than did ruminal supplementation.

In general, the effects of ruminal/abomasal supplementation were between those of ruminal supplementation and those of abomasal supplementation. The intermediate RAN concentration (Table 4) and PDI (Table 5) provided animals with the previously discussed effects of body nitrogen retention, providing NB that was similar to the exclusive rumen or abomasum supplementation (Table 5).

From the results obtained in the present study, it can be stated that nitrogen supplementation in the rumen or abomasum should show similar effects but as a result of different ways to improve nitrogen status in the animal metabolism. In theory, these mechanisms provide a possibility for choosing between supplementation with RDP or RUP.

Consequently, additional factors should be considered with regard to the most suitable production system. The costs involved in using RDP sources are almost always lower than those of RUP. Considering that the primary mechanism for increasing nitrogen retention in the body using RDP is an overall improvement in dietary nitrogen availability, the use of non-protein nitrogen sources, such as urea, could reduce costs compared with the use of RUP. In addition, the responses to RUP supplementation for increased metabolizable protein supply depend on AA profile of the protein source, which must be compatible with the profile required by the animal anabolism. Therefore, even if the source of RUP is inexpensive, the most appropriate formulas would depend on the profile of digestible AA in the small intestine.

Consequently, supplementation with RUP sources are only feasible or logical after the beneficial effects of RDP had been explored, thereby providing additional performance gains through direct supply of metabolizable protein [8, 20].

## Conclusion

The supplementation of cattle fed tropical forage with protein in the rumen, abomasum, or both can increase the retention of nitrogen in animal. However, the metabolic pathways involved in improving nitrogen accretion differ between supplementation sites. The improvement obtained with rumen supplementation seems based on a direct increase in dietary nitrogen availability and status. Moreover, the improvement obtained with abomasum supplementation results from an increased supply of metabolizable protein.

## Abbreviations

3-MH: urinary excretion of 3-methyl histidine
AA: amino acids
BW: body weight
CP: crude protein
DM: dry matter
DMF: dry matter from forage
DNDF: digested neutral detergent fiber
DOM: digested organic matter
iNDF: indigestible neutral detergent fiber
ki kp and kd: rates of intake, passage and degradation of NDF
NB: nitrogen balance
NBR: nitrogen balance in the rumen
NDF: neutral detergent fiber
NDFap: neutral detergent fiber assayed with a heat-stable alpha-amylase and corrected for contaminant ash and protein
OM: organic matter
PDI: protein digested in the intestine
RAN: rumen ammonia nitrogen
RDP: rumen degradable protein
RUP: rumen undegradable protein
SUN: serum urea nitrogen
VFA: volatile fatty acids
